# A Registration Method Based on Contour Point Cloud for 3D Whole-Body PET and CT Images

**DOI:** 10.1155/2017/5380742

**Published:** 2017-02-21

**Authors:** Zhiying Song, Huiyan Jiang, Qiyao Yang, Zhiguo Wang, Guoxu Zhang

**Affiliations:** ^1^Software College, Northeastern University, Shenyang 110819, China; ^2^Department of Nuclear Medicine, General Hospital of Shenyang Military Area Command, Shenyang 110840, China

## Abstract

The PET and CT fusion image, combining the anatomical and functional information, has important clinical meaning. An effective registration of PET and CT images is the basis of image fusion. This paper presents a multithread registration method based on contour point cloud for 3D whole-body PET and CT images. Firstly, a geometric feature-based segmentation (GFS) method and a dynamic threshold denoising (DTD) method are creatively proposed to preprocess CT and PET images, respectively. Next, a new automated trunk slices extraction method is presented for extracting feature point clouds. Finally, the multithread Iterative Closet Point is adopted to drive an affine transform. We compare our method with a multiresolution registration method based on Mattes Mutual Information on 13 pairs (246~286 slices per pair) of 3D whole-body PET and CT data. Experimental results demonstrate the registration effectiveness of our method with lower negative normalization correlation (NC = −0.933) on feature images and less Euclidean distance error (ED = 2.826) on landmark points, outperforming the source data (NC = −0.496, ED = 25.847) and the compared method (NC = −0.614, ED = 16.085). Moreover, our method is about ten times faster than the compared one.

## 1. Introduction

The fusion image of Positron Emission Tomography (PET) and Computed Tomography (CT) provides the functional and metabolic information, which could help medical diagnosis, treatment planning, and evaluation [1, 2]. Because of the different scanning time of PET and CT and the respiratory movements, there are always image artifacts and deformations existing in PET/CT images, bringing enormous challenges to the research work on image fusion. Therefore, it is necessary to find an efficient and accurate registration method to improve the correspondence between PET and CT images before fusion, which is also of great significance to clinical diagnosis and treatment [3, 4].

Image registration is the process of aligning one image (moving image) to another image (fixed image) by finding a transformation that maximizes the similarity metric between the transformed moving image and the fixed image [5]. The registration of PET and CT images is a matter of multimodal image registration. Recently, there are many methods proposed in the field of image registration. They are generally classified into two categories: intensity-based and feature-based methods.

As for intensity-based methods, [6] proposed Mattes Mutual Information (MMI) as a similarity metric for PET-CT image registration in the chest. The study in [7] combined the Harris corners as the feature information to enhance the conventional MMI registration. A registration method based on multiresolution generic algorithms [8] for 3D registration of cardiac PET/CT images achieved a high accuracy in a reasonable time. The study in [9] combined the segmentation and intensity information together for PET/CT registration, whose speed and accuracy were enhanced. As for feature-based methods, [10] presented a preprocessing method for MRI and CT images and a feature-based affine transformation where tibia and femur were selected as the feature for the registration of human knee images. Reference [11] modified Iterative Closest Points (ICP) by combining moments, center points, and Canny calculators, which had a good result on head images with a high speed. Reference [12] presented a registration framework based on the geometrical characters for aligning CT data that reduced the processing time heavily without affecting the registration accuracy.

However, the accuracy and time efficiency of 3D registration on whole-body PET and CT images are still challenging. The intensity-based methods often have a higher accuracy but less time efficiency because of the large computation, while the feature-based methods with less computation are faster but the accuracy depends heavily on the extracted feature [13–16]. To address these issues, this paper presents a multithread registration solution based on contour point cloud for 3D whole-body PET and CT images. There are mainly three contributions in this paper. Firstly, a geometric feature-based segmentation (GFS) method and a dynamic threshold denoising (DTD) method are creatively proposed for removing background noise in CT and PET images, respectively. Secondly, this paper proposes a new automated trunk slices extraction method to extract feature point cloud sequences automatically. This method is different from the manual method proposed in [17] where the identification of trunk slices must be done manually. Finally, a simple downsampling method and a multithread ICP are used to achieve registration quickly with a considerable accuracy.

The remaining sections of this paper are organized as follows.  Section 2 presents our proposed registration method. In Section 3, we introduce a new geometric feature-based segmentation method and dynamic threshold denoising method to preprocess the CT and PET images, respectively. The feature point cloud extraction is shown in Section 4.  Section 5 explains the multithread ICP in detail. We compare our proposed method with one commonly used registration method and the experimental results evaluation and discussion are shown in Section 6. Finally, the conclusions and future work are given in Section 7.

## 2. Registration Method and Steps

**Figure 1:**
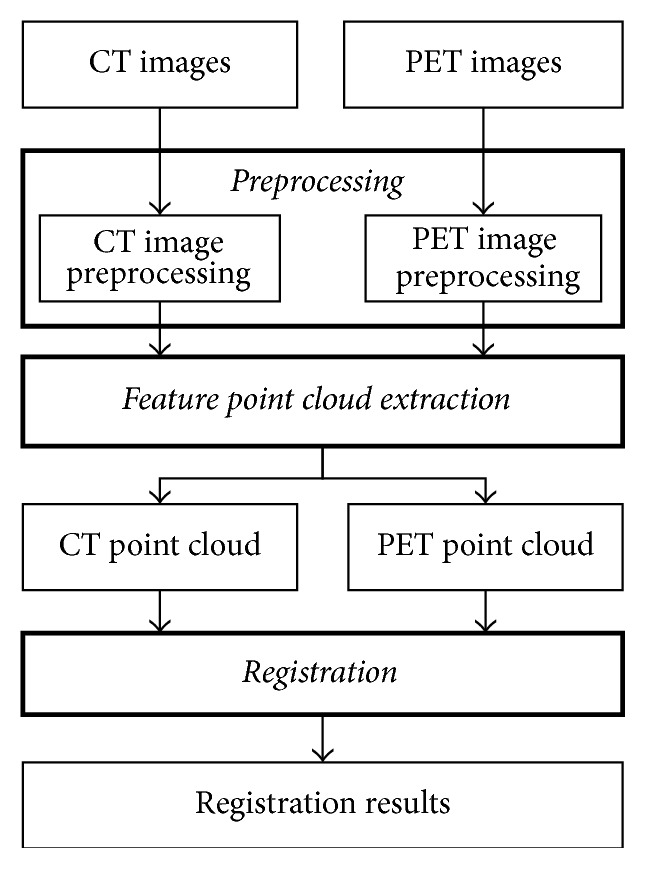
The flowchart of the proposed registration method.

To address the challenges and requirements mentioned above with considerable accuracy and efficiency, this paper proposed a multithread registration solution based on contour point cloud for PET and CT images.  Figure 1 is the flowchart of the proposed method where the bold rectangles mean our innovation works. The inputs, CT and PET images, are the original image sequences in DICOM format from the same patient. The point cloud is the set of feature points used as the inputs of the registration.

**Figure 2:**
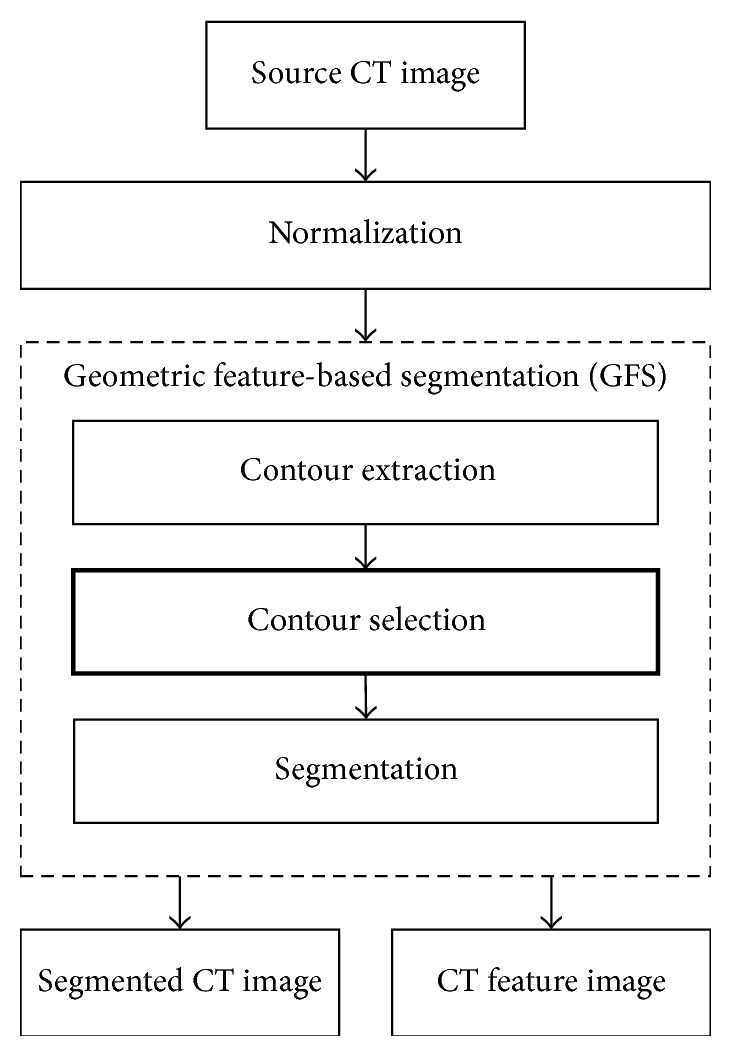
The flowchart of CT image preprocessing.

**Figure 3:**
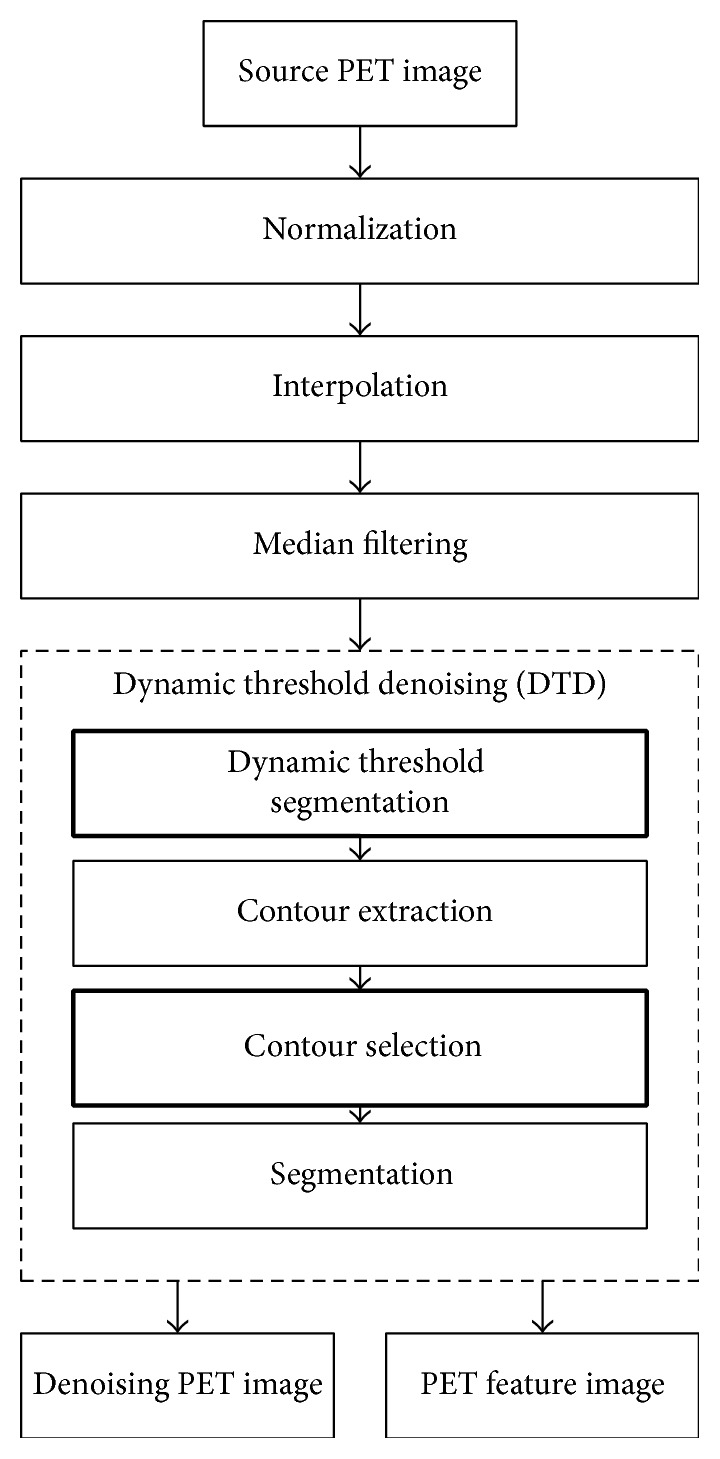
The flowchart of PET image preprocessing.

As shown in Figure 1, the proposed method contains three phases: preprocessing, feature point cloud extraction, and registration. Firstly, the preprocessing includes two parts, CT image preprocessing and PET image preprocessing. Figures 2 and 3 are the flowcharts of them, respectively. In this phase, we primarily perform normalization that maps intensities of both PET and CT images to the range of [0,255]. Besides, in PET preprocessing, a cubic b-Spline interpolation is employed to interpolate the PET images from 128 × 128 to the same resolution as CT images (512 × 512). Next, the GFS method and DTD method are utilized on CT and PET images, respectively, in order to get segmented images and binary feature images. The GFS method combines the slice id and contour selection together to eliminate background voxels of CT images (e.g., the shadow of scanning bed). The DTD method integrates a dynamic threshold segmentation and contour selection to get high quality PET images with little noise. After the preprocessing, we use a new automated trunk slices extraction method according to our definition of trunk slices (see Section 4) to identify trunk slices from the obtained feature images and then extract feature point clouds from them. In the last phase, a simple downsampling method is primarily used on the extracted feature point clouds before registration to reduce the computation. Then, a multithread ICP is adopted to obtain the registration results where multithreads could further enhance the time efficiency.

## 3. Preprocessing with GFS and DTD 

**Figure 4:**
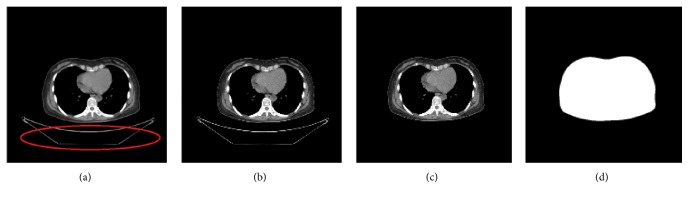
An example of CT preprocessing results. (a) The source CT image. (b) The normalization result. (c) The final preprocessing result (segmented result). (d) The feature image.

**Figure 5:**
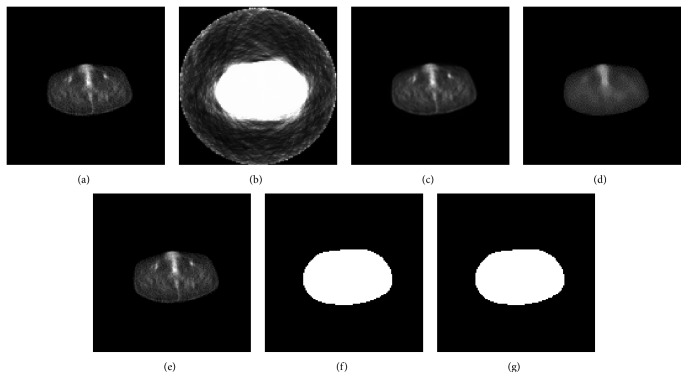
An example of PET preprocessing results. (a) The source PET image (128 × 128). (b) is got by applying binary threshold segmentation on (a). (c) The interpolation result (512 × 512). (d) The median filtering result. (e) The final preprocessing result. (f) is got by applying a binary threshold segmentation on (e). (g) The feature image obtained.

It is desirable to do preprocessing before registration due to the following reasons [18]. Firstly, intensity normalization is needed because the intensity range of the original CT and PET images is too wide that would cause high computational complexity. Secondly, the resolutions of PET and CT images are different so that an interpolation should be performed on PET images. Finally, it is necessary to do segmentation to remove the massive background noise information (the pixels do not belong to human body) in both CT and PET images because that could affect the registration accuracy heavily. For example, the noise in CT images (see the red circle in Figure 4(a)) is from the shadow of the scanning bed. The noise captured from the background in PET image (see Figure 5(a)) is hard to be observed due to its low value of intensity. In order to observe the noise in PET directly, we perform binary threshold segmentation on Figure 5(a). The threshold segmentation result is shown in Figure 5(b), where the isolated points are the noisy pixels.

Different preprocessing methods are proposed for CT and PET images because of the different kinds of noise. Figures 2 and 3 are the flowcharts of CT and PET image preprocessing, respectively, where bold rectangles are the innovative works of this paper. After preprocessing, the segmented results of PET and CT images and the feature images used for feature point extraction could be obtained. The segmented results of PET and CT would be as the moving and fixed images in registration, respectively. In this paper, the outlines of the human body are the anatomical feature that we focus on because only pixels of the human body make sense to clinical process. The feature image is defined as a binary image where the intensity values of all the pixels inside the outlines of the human body are set to 255 and the rest of pixels outside the human body are set to 0. According to these feature images, we can identify and remove those pixels that do not belong to human body and then obtain the segmented result.

### 3.1. CT Image Preprocessing

#### 3.1.1. Normalization of the CT Image

To normalize the intensity of a source CT image to the range of [0,255], the following formula is used:

(1) $$\begin{array}{c} I^{\prime }\left(\;x\;\right)=\frac{I\left(x\right)-I_{\min}}{I_{\max}-I_{\min}}\times 255, \end{array}$$

where [*I*_min_, *I*_max_] is the intensity range of an input image. For CT images, *I*_max_ = wc + ww/2.0 and *I*_min_ = wc − ww/2.0. wc and ww are window center and window width provided in DICOM data.

#### 3.1.2. Geometric Feature-Based Segmentation (GFS)

This paper proposes a GFS method (see Algorithm 1) that combines the id of slices in image sequences and geometric characteristics together to remove the background noise in CT images and obtain segmented images and feature images. Algorithm 1 contains three phases. Firstly, step (1) means contour extraction where all the external contours (CL) of the input image are detected here and then sorted in descending order according to their area. Secondly, steps (2)–(22) are the contour selection procedure. In step (2), those contours with area less than 1000 pixels are removed. The number of the remaining contour list (CL) is defined as *h*. After that, in steps (3)–(22), we select those contours that could represent the outlines of human body based on the relationships among *h*, the area value of the contours, and the slice id of the input feature image. The size means the number of elements contained by the selected effective contour list (ECL). Finally, the rest of steps (23)-(24) are the segmentation phase. In step (23), the feature image (*F*) could be obtained by setting the intensity value of all the pixels inside the contours of ECL to 255 and setting the rest of pixels to 0. Besides, the mask image (*M*) is similar to the feature image except that the pixels inside the contours of ECL are set to 1. Then, in step (24), by multiplying the input image (*S*) with the mask image (*M*), the segmented result (*O*) could be obtained where the background noise in the source CT image has been removed.

An example of CT preprocessing results is shown in Figure 4. Figure 4(a) is one source CT image as an input image. The red circle signs the background noise in the CT image needed to be removed. Figures 4(b), 4(c), and 4(d) are the normalization result as the input image of GFS, the final preprocessing result (the segmented result) as the fixed image in registration, and the feature image for extracting feature points, respectively. It is clear that the background noise has been removed totally after preprocessing.

### 3.2. PET Image Preprocessing

#### 3.2.1. Cubic B-Spline Interpolation

Similarly, we firstly perform normalization on PET images according to formula (1). Then, the function, BSplineInterpolateImageFunction, provided by Insight Segmentation and Registration Toolkit (ITK) is employed to interpolate PET images from 128 × 128 to the same resolution as CT images (512 × 512).

#### 3.2.2. Median Filtering

The median filter is commonly used to replace the intensity value of each pixel with the median of neighboring values, which could not only eliminate the isolated noisy points but also preserve the edges well with less blur problem. As a result, we adapt median filter to remove single point noises of PET images.

#### 3.2.3. Dynamic Threshold Denoising (DTD)

As shown in Figure 3, the DTD method combines the dynamic threshold segmentation, contour extraction, contour selection, and segmentation sequentially to achieve noise elimination of PET images and then obtain denoising images (segmented results) and feature images used for image registration and feature point extraction, respectively. Due to the different intensity distribution in different slices, dynamic threshold segmentation is performed on images after the median filtering. The threshold is dynamically determined by the peak of the gray histogram and the id of the current slice in the image sequences.  Algorithm 2 shows the detailed steps of the dynamic threshold segmentation. Firstly, the gray histogram (*H*) of the input image (*S*) is calculated. After that, the gray value (peakLoc) corresponding to the peak of the histogram could be obtained in step (2). Then, in steps (3)–(7), the threshold used in threshold segmentation (in step (8)) is calculated by estimating the relationships between the slice id and the peakLoc. Finally, the result of dynamic threshold segmentation (*T*) could be obtained in the last step (8). Note that the procedures of contour extraction, contour selection method, and the final segmentation employed in DTD are similar to those utilized in CT preprocessing.

Figure 5 illustrates an example of PET preprocessing results. Figure 5(a) is the source PET image (128 × 128). Figures 5(c) and 5(d) are the interpolation result (512 × 512) and median filtering result, respectively.  Figure 5(e) is the final processing result (the segmented result) which would be as a moving image in registration.  Figure 5(g) is the feature image obtained by DTD. Due to the low value of the intensity, the noisy points in Figure 5(a) are unable to be observed directly. We perform binary threshold segmentation on both Figures 5(a) and 5(e) in order to illustrate the performance of noise elimination intuitively (Figure 5(e) is an image with less noisy pixels compared to Figure 5(a)). The threshold chosen here is 0, which means that the pixels whose intensity value is bigger than 0 are set to 255, while the other pixels are set to 0. Their threshold segmentation results which highlight the noisy points are shown in Figures 5(b) and 5(f), respectively. It is clear that the small and isolated points (noisy pixels) have been effectively removed by comparing Figures 5(b) and 5(f). This approves that the proposed DTD method could reduce noise in PET effectively.

## 4. Feature Point Cloud Extraction

The points on the outlines of one feature image are defined as the feature points for one specific slice. A feature point cloud is the set of the feature points contained by one specific feature image. It could be obtained by writing the coordinates of one set of feature points into a corresponding point cloud file. The feature point cloud sequences for CT or PET data are constructed by all of the point cloud files extracted from the corresponding feature image sequences, which would be as the input data of the registration.

**Figure 6:**
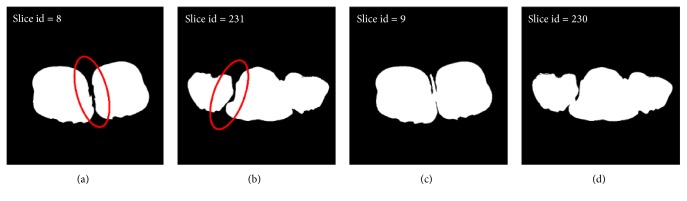
The illustration of trunk slices. (a) and (b) are not trunk slices. (c) and (d) are trunk slices. The slice id means the serial number of one image in the slice sequences. Red circle represents separated regions.

However, there is some useless information in the input clinical slices. For example, some slices that include only arms and legs of a human body have little clinical meaning for a doctor. Therefore, in order to reduce the computation and increase the time efficiency, a new automated trunk slices extraction method is proposed in this paper which is different from the manual method in [17]. Trunk slices are considered as those slices that contain only one connected region. Following this definition, we can traverse the feature image sequences and calculate the number of connected regions for every image. If the number of connected regions is equal to one, then the slice is regarded as a trunk slice. As shown in Figure 6, Figures 6(a) and 6(b) are not trunk slices, which contained more than one connected region (see red circle), while Figures 6(c) and 6(d) are trunk slices.

The PET and CT slice sequences and the feature image sequences of one specific data pair are in correspondence one by one, so the trunk slices in PET could be determined as long as the trunk slices in CT are found. After extracting trunk slices, the feature point clouds can be obtained automatically by writing the coordinates of the feature points of every feature image into a corresponding point cloud file. The detailed steps of the feature point cloud extraction are shown in Algorithm 3. Firstly, the number of connected regions (NCR) for every image in the feature image sequences (FIS) is calculated in step (1). Next, in steps (2) and (3), the slice id of the first and the last slice with only one connected region (*M*1 and* M*2) can be obtained by traversing NCR. Finally, in step (4), the feature points of all the feature images between* M*1 and* M*2 are extracted and then the coordinates of these feature points are written into the corresponding point cloud file.

## 5. Registration

Generally, ICP could be adopted on the input feature point clouds to calculate the parameters of an affine transformation matrix. Then the matrix is used to transform the moving images and then obtain the registration results. However, the extracted feature point clouds are still large for registration, so we use a simple downsampling method before registration to reduce the computation and increase the time efficiency. Subsequently, a multithread ICP is employed for registration to further enhance the time efficiency.

### 5.1. Downsampling

**Figure 7:**
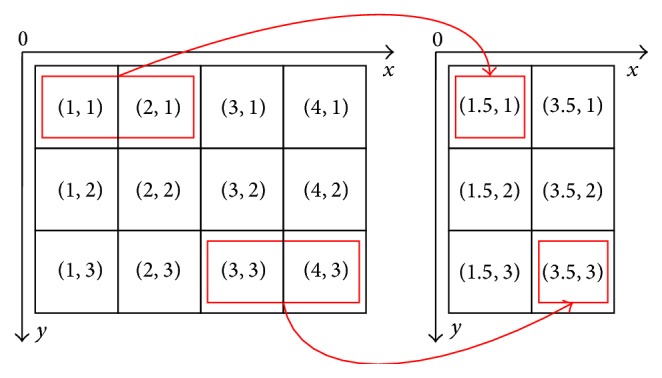
An example of downsampling with *α* = 2 in *x* direction.

Firstly, a sample ratio *α* (a positive integer) and a downsampling direction (*x* or *y*) should be determined. Then, the average coordinate of *α* adjacent points along the selected direction is calculated as the new feature point. Note that each original feature point is only used one time.  Figure 7 illustrates an example that is downsampling with *α* = 2 in *x* direction. With the increase of *α* and the number of downsampling directions, the registration speed will be greatly enhanced but the registration accuracy will be decreased heavily. Through a lot of experiments, we find that the accuracy and speed are both pretty good while downsampling in single direction and *α* = 50.

### 5.2. Multithread ICP

ICP, first proposed in [19], is widely employed on the registration of two point clouds. ICP iteratively revises the transformation to minimize the distance (cost function) between the fixed and moving point clouds through an optimizer [12]. The cost function and optimizer used in the traditional ICP are mean squared error and iterative least-squares approach, respectively. Firstly, ICP finds the closest point in the moving point cloud for every point in the fixed point cloud. Then, the transformation matrix is calculated for every pair of points. Next, the corresponding points found in the previous step could be matched precisely by finding the transformation matrix based on the cost function. After that, the moving points could be transformed by using the obtained matrix. Finally, the former steps are iterated until satisfying certain criteria.

Parallel computing is an effective way to improve the processing power and computing speed. To enhance the time efficiency of registration, in this paper, a multithread (4 threads are used) ICP method is employed. Different from the traditional ICP, we use the Euclidean distance and Levenberg Marquardt algorithm [20] as the cost function and optimizer, respectively. By the multithread ICP, we can calculate the transformation matrix and guide the affine transformation. The process of applying an affine transform to a point in 3D space is shown in the following formula:

(2) x′y′z′=M00M01M02M10M11M12M20M21M22·x−Cxy−Cyz−Cz+Tx+CxTy+CyTz+Cz,

where 3 × 3 matrix $M=\left[\begin{array}{ccc} M_{00} & M_{01} & M_{02}\\ M_{10} & M_{11} & M_{12}\\ M_{20} & M_{21} & M_{22} \end{array}\right]$ represents rotations, anisotropic scaling, and shearing and *C* = (*C*_*x*_, *C*_*y*_, *C*_*z*_)^*T*^ and *T* = (*T*_*x*_, *T*_*y*_, *T*_*z*_)^*T*^ are the rotation center and translation coefficients. (*x*, *y*, *z*)^*T*^ and (*x*′, *y*′, *z*′)^*T*^ are the source point and the transformed point, respectively.

Briefly, the procedure of the multithread ICP is as follows. The input data of the multithread ICP are the extracted feature point clouds of PET and CT that are as the moving point cloud and fixed point cloud, respectively. Firstly, the input data are divided into four groups. Then, the four groups of data are computed by ICP algorithm in parallel on different threads. Finally, an affine transformation is performed on the moving image sequences (the segmented results of PET image sequences) after calculating the transformation matrixes for all of the slices. And then, the registration results could be obtained.

## 6. Results and Discussion

### 6.1. Experimental Data and Platform

The experimental data are provided by the General Hospital of Shenyang Military Area Command, Shenyang, China. There are 13 pairs of PET/CT data used and the detailed information is shown in Table 1.

The experimental platform is Intel® Core™ i7-2600 CPU @ 3.40 GHz, 8 G RAM, 1T hard disk, Windows 7 OS. The integrated development environment is Visual Studio 2013 and C++ is the only one programming language adopted in this paper. Besides, there are several open source libraries employed including OpenCV, ITK, Visualization Toolkit (VTK), and Point Cloud Library (PCL). The ITK-SNAP and Mango are two applications utilized to view images.

### 6.2. Registration Accuracy Measure

To quantitatively evaluate the registration accuracy of the proposed method and the compared method, two metrics are applied in this paper including negative normalized correlation metric (NC) and the Euclidean distance error (ED) of the landmark points [21]. These measures are defined as follows:

(3) NCA,B=−1×∑i=1NAi·Bi∑i=1NAi2∑i=1NBi2,EDFSA,FSB=1N∑i=1Ndpointi,

where *A*_*i*_ and *B*_*i*_ are the *i*th pixel of the binary feature images *A* and *B*, respectively. *FS* means the landmark points of one input image. *d*(point_*i*_) represents the minimum distance from each point of *FS*_*A*_ to *FS*_*B*_. Note that the range of NC is [−1,0].

These two metrics measure the degree of difference between two input images from different perspective. NC calculates the normalized negative pixel-wise cross-correlation of the 3D correspondence binary feature images to access the warp of the entire input images. There are two reasons of evaluating the binary feature images instead of source images. Firstly, the outlines of human body are the features that we focus on as only the pixels of human body have clinical meaning to doctors. Secondly, the PET images and CT images are different so that it is easier to evaluate the feature images with the positional information. Moreover, we choose all of the feature points of the source images as the landmark points to evaluate the registration accuracy. ED calculates the mean Euclidean distance error on the corresponding feature points of the input images to access the positional correspondence of these points. Poor matching between two images* A* and* B* denotes large values of NC and ED.

### 6.3. Results Evaluation and Discussion

The speed and the accuracy are the main factors to evaluate the performance of a registration method [5]. With respect to these two factors, several experiments are conducted on 13 pairs of data. Additionally, the superiority of the proposed registration method is assessed by comparing its results with another method which is abbreviated as MR + GD + MMI in this paper. MR + GD + MMI applies gradient descent algorithm (GD) [22] and MMI [6] as the optimizer and the similarity metric, respectively. It is a multiresolution (MR) [8] registration method based on affine transformation. There are two reasons of comparing the performance with MR + GD + MMI. Firstly, MMI is often used for multimodal registration and has a relatively high accuracy and speed [23]. Secondly, a MR based registration approach is widely used to improve the speed, accuracy, and robustness of registration. The experimental data and accuracy evaluation metrics are shown in Sections 6.1 and 6.2, respectively. The results and comparisons with MR + GD + MMI are discussed as follows.

**Figure 8:**
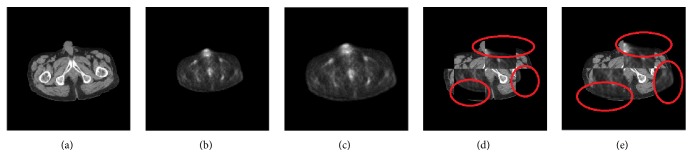
An example of the registration results. (a) A CT image (fixed image). (b) A PET image (moving image). (c) The registration result from (b) to (a). (d) A checkerboard composite of (a) and (b). (e) A checkerboard composite of (a) and (c).

Figure 8 illustrates one case of our proposed method results. Figures 8(a) and 8(b) are the segmented results of CT preprocessing and PET preprocessing, respectively.  Figure 8(a) is as the fixed image and Figure 8(b) is as the moving image.  Figure 8(c) is the registration result by aligning Figures 8(b) and 8(c).  Figure 8(d) presents a checkerboard composite of the fixed image (Figure 8(a)) and the moving image (Figure 8(b)).  Figure 8(e) shows a checkerboard composite of the fixed image (Figure 8(a)) and the registration result (Figure 8(c)) after registration. Obviously, the correspondence between the outlines of PET and CT images is increased dramatically after registration (e.g., the regions marked by red circles in Figures 8(d) and 8(e)), which demonstrates the effectiveness of our proposed method.

**Figure 9:**
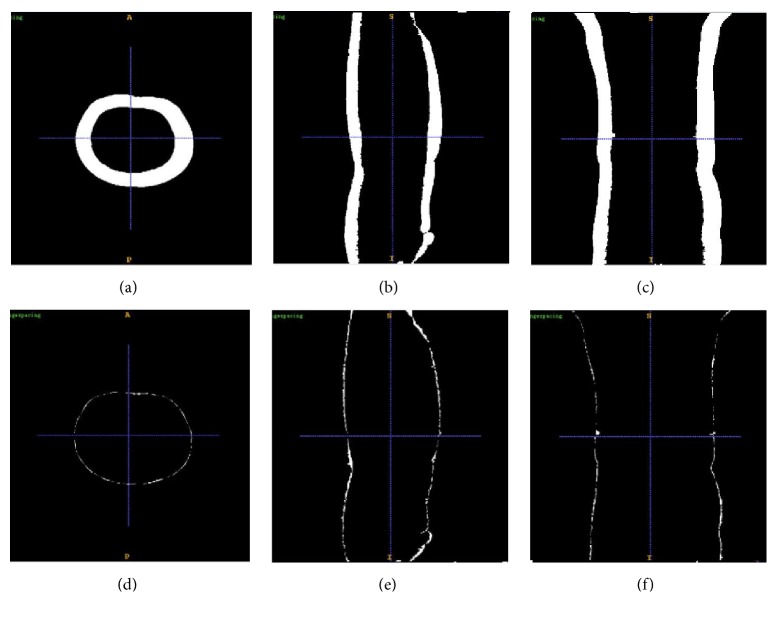
An example of the subtraction of PET and CT feature image sequences. (a), (b), and (c) represent the subtraction before registration and (d), (e), and (f) represent the subtraction after registration. (a) and (d) are along the transverse plane, (b) and (e) are along the sagittal plane, and (c) and (f) images are along the coronal plane.

Moreover, we reconstruct the subtraction of PET and CT feature image sequences in order to explain the registration result more intuitively. An example is shown in Figure 9 where the images in (a), (b), and (c) show the difference before registration and those in (d), (e), and (f) correspond to the difference after registration. Figures 9(a) and 9(d) are along transverse plane, Figures 9(b) and 9(e) are along sagittal plane, and Figures 9(c) and 9(f) are along coronal plane. From Figure 9, it can be easily observed that the outlines of PET and CT intertwine each other with much slighter variance after registration. Hence, through subjective evaluations of our experiments results, our proposed method could achieve a good result on 3D whole-body PET and CT images.

Further, to support these subjective evaluations of the proposed method, quantitative analysis is also conducted in this paper. We compare the accuracy measures (NC and ED) of the source data with the registration results. The details of the comparisons are listed in Table 2. Note that the smaller values of NC and ED, the higher similarity of two images, the higher registration accuracy. From Table 2, it can be seen that both the values of NC and ED of all experimental data are decreased after registration which demonstrates the effectiveness of these two examined methods. In addition, the average values of these two metrics of our method (NC = −0.93289 and ED = 2.82614) are better than the compared method (NC = −0.61424 and ED = 16.08516), which proves that our proposed method outperforms MR + GD + MMI in terms of registration accuracy. Moreover, our method has a better stability than MR + GD + MMI because the standard deviations of NC and ED (0.02341 and 1.23775, resp.) are smaller than the standard deviations of the compared one (0.179473 and 12.23811, resp.).

This accuracy improvement was due to the feature point clouds extracted from the PET and CT images employed in our proposed method. Specifically, the tight positional correspondence between these two point clouds could ensure the registration accuracy and stability. However, the metric MMI used in MR + GD + MMI only makes a fairly loose assumption that image intensities should have a probabilistic relationship and MMI based registration could fail when statistical dependence between images is weak [23]. Because of the different modality of the PET and CT images, it would sometimes affect the performance of MMI so as to decrease the registration accuracy of the compared method.

Finally, a comparative study on computational time has been done to explore the efficiency of the proposed method and the compared method. From Table 3, the average processing time of our method is 2174 s, which is only about 9.25% of the time taken by MR + GD + MMI (23511 s). MR + GD + MMI is time-consuming because each calculation for MMI needs to involve all the pixels of the input images and the number of iterations during optimization is very large. However, our registration method only involves a certain number of feature points not all the pixels of the input images so as to enhance the time efficiency. Specifically, in our proposed method, we also use a new automated truck sliced extraction method to reduce unimportant information (such as the slices containing only legs or arms), a simple downsampling method to further decrease the number of feature points used for registration, and a multithread ICP to do computing work in parallel for further reducing the computational amount as well as the processing time. In summary, the proposed method has a higher accuracy and better efficiency than MR + GD + MMI.

## 7. Conclusion and Future Work

In this paper, a multithread ICP method based on contour point cloud is proposed for registration of 3D whole-body PET/CT images which has a great value to PET/CT fusion [24]. Firstly, to eliminate background noise in the PET and CT images and increase registration accuracy, a geometric feature-based segmentation method and a dynamic threshold denoising method are presented for CT and PET preprocessing, respectively. Then, we put forward a new automated trunk slices extraction method to extract the feature point cloud. Next, to further enhance the time efficiency, a simple downsampling method is performed before registration to reduce the computation. Finally, a multithread ICP solution is used to guide the affine transformation and then achieve the PET/CT registration with a higher speed. To verify the effectiveness of our proposed method, we perform the registration experiments on 13 pairs (246~286 slices per pair) of PET and CT data. The numerical results and comparisons with MR + GD + MMI prove that our proposed method has a higher accuracy and speed.

However, there are two weaknesses in our proposed method. Firstly, the application range is limited because some steps of our method are designed only for whole-body PET and CT. Secondly, although this paper uses a multithread ICP based on parallel-computation to reduce registration time, the processing time could still be enhanced. In the future, we would try our proposed method on GPU to further improve the performance.

## Figures and Tables

**Algorithm 1:**
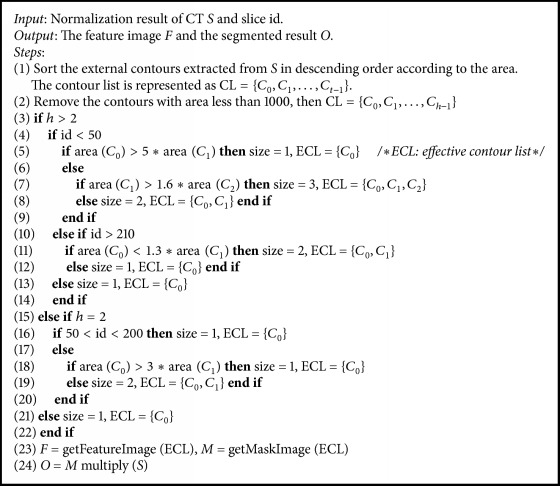
Geometric feature-based segmentation (GFS).

**Algorithm 2:**
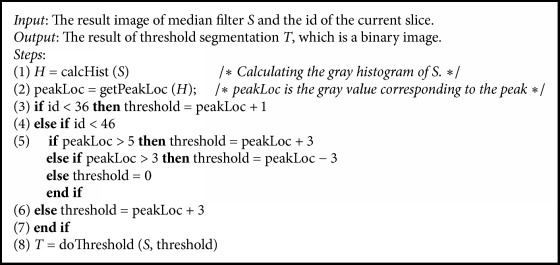
Dynamic threshold segmentation.

**Algorithm 3:**
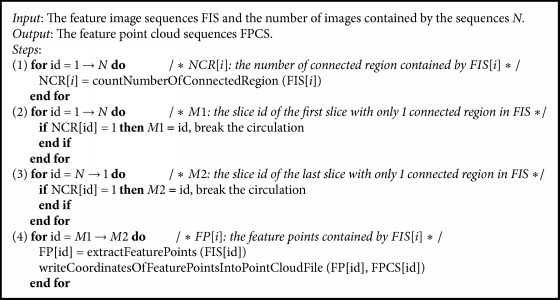
Feature point cloud extraction.

**Table 1:** The detailed information of PET/CT data adopted in experiment.

Attribute	PET	CT
Image matrix size	128 × 128 × 246~286	512 × 512 × 246~286
Pixel size	5.469 × 5.469 mm^2^	0.977 × 0.977 mm^2^
Slice thickness	3.270 mm	3.270 mm
The number of trunk slices	194~226	194~226

**Table 2:** The comparisons of two similarity metrics on 13 pairs of PET and CT data.

ID	Methodology					
	Source data		The proposed method		MR + GD + MMI	
	NC	ED	NC	ED	NC	ED
1	−0.50279	23.6101	−0.95232	1.87713	−0.90144	3.92457
2	−0.48013	30.0264	−0.95906	1.77737	−0.56848	19.6972
3	−0.52668	22.2486	−0.87322	6.35008	−0.73011	13.1091
4	−0.50683	23.8465	−0.95017	2.02653	−0.24907	52.8046
5	−0.49865	28.7776	−0.91258	4.09511	−0.57158	13.3536
6	−0.49636	25.7057	−0.93776	2.5987	−0.69698	7.43801
7	−0.47822	26.9906	−0.92210	3.01465	−0.52484	16.4022
8	−0.48632	26.5894	−0.93850	2.53644	−0.51073	14.3721
9	−0.48787	27.2716	−0.94317	2.16966	−0.61221	19.2727
10	−0.49576	23.6947	−0.90799	3.37168	−0.46089	15.2446
11	−0.49330	26.1713	−0.94317	2.34775	−0.52306	18.9303
12	−0.49176	26.4674	−0.94490	2.22978	−0.89865	4.13041
13	−0.50106	24.6159	−0.94265	2.34491	−0.73704	10.4277

*Average*	*−0.49583*	*25.8474*	*−0.93289*	*2.82614*	*−0.61424*	*16.08516*

*Standard deviation*	*0.01255*	*2.2090*	*0.02341*	*1.23775*	*0.179473*	*12.23811*

**Table 3:** The comparisons of processing time on 13 pairs of PET and CT data.

ID	The number of trunk slices	The proposed method	MR + GD + MMI
1	202	1373 s	9581 s
2	219	2537 s	21676 s
3	208	1527 s	11410 s
4	208	1510 s	29149 s
5	226	3704 s	31169 s
6	221	2801 s	30140 s
7	194	1764 s	30358 s
8	209	2612 s	25919 s
9	199	2488 s	42415 s
10	218	1719 s	30085 s
11	208	1608 s	27403 s
12	211	2930 s	8721 s
13	213	1697 s	7627 s

*Average*	*210*	*2174 s*	*23511 s*
